# Capturing the experiences of patients with inherited optic neuropathies: a systematic review of patient-reported outcome measures (PROMs) and qualitative studies

**DOI:** 10.1007/s00417-021-05534-0

**Published:** 2022-01-13

**Authors:** Benson S. Chen, Tomasz Galus, Stephanie Archer, Valerija Tadić, Mike Horton, Konrad Pesudovs, Tasanee Braithwaite, Patrick Yu-Wai-Man

**Affiliations:** 1 John Van Geest Centre for Brain Repair and MRC Mitochondrial Biology Unit, Department of Clinical Neurosciences, University of Cambridge, Cambridge, UK; 2 Cambridge Eye Unit, Addenbrooke’s Hospital, Cambridge University Hospitals, Cambridge, UK; 3 Primary Care Unit, Department of Public Health and Primary Care, University of Cambridge, Cambridge, UK; 4 School of Human Sciences, University of Greenwich, London, UK; 5 Psychometric Laboratory for Health Sciences, University of Leeds, Leeds, UK; 6 School of Optometry and Vision Science, University of New South Wales, Kensington, Australia; 7 School of Life Course Sciences, King’s College London, London, UK; 8 The Medical Eye Unit, Guy’s and St Thomas’ NHS Foundation Trust, London, UK; 9 Moorfields Eye Hospital NHS Foundation Trust, London, UK; 10 Institute of Ophthalmology, University College London, London, UK

**Keywords:** Leber hereditary optic neuropathy, Dominant optic atrophy, Quality of life, Patient-reported outcome measure

## Abstract

**Purpose:**

To identify and comprehensively evaluate studies capturing the experience of individuals affected by an inherited optic neuropathy (ION), focusing on patient-reported outcome measures (PROMs) and qualitative studies where the health status and quality of life (QoL) of these individuals have been explored.

**Methods:**

Systematic review of five databases using a search strategy combining four concepts: (1) ION; (2) QoL and health status; (3) PROMs; and (4) qualitative research. Studies assessing the impact of ION on any QoL domain using a PROM or qualitative methodology were included and appraised, using criteria based on the COSMIN checklist (for PROM studies) and the CASP checklist (for qualitative studies).

**Results:**

Of 1326 unique articles identified, six studies were included. Five PROMs were identified: Visual Function Index (VF-14); Hospital Anxiety and Depression Scale (HADS); a novel graphical online assessment tool (NGOAT) for reporting emotional response to vision loss; a new PROM informed by the DSM-V Criteria for Major Depressive Disorder; and an interpersonal and career ‘impact rating’ PROM. The psychometric performance of included PROMs were poorly described. Qualitative studies found that vision loss resulted in psychosocial losses including loss of social and communication skills and loss of independence and freedom. Factors that modified the response to vision loss were also identified.

**Conclusion:**

The current PROMs used by individuals with ION have poor content coverage, primarily measuring activity limitation and emotional well-being, and insufficient reporting of psychometric performance. There is a need to develop a PROM for individuals ION to report their experiences of living with their condition. 
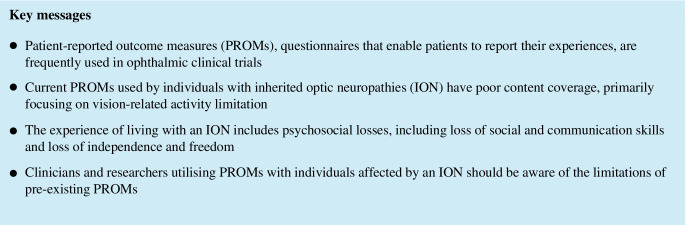

**Supplementary Information:**

The online version contains supplementary material available at 10.1007/s00417-021-05534-0.

## Introduction

Inherited optic neuropathies (IONs) constitute a genetically and clinically heterogeneous group of rare disorders that result in progressive optic nerve degeneration and irreversible visual loss [1, 2]. Autosomal dominant optic atrophy (DOA) and Leber hereditary optic neuropathy (LHON) are the two most common IONs encountered in clinical practice, with an estimated population prevalence of 4–10 per 100,000 individuals and 2–3 per 100,000 individuals, respectively [2]. The pathological hallmark of IONs is the preferential loss of retinal ganglion cells, which results in a central pattern of visual loss. Individuals with DOA usually become symptomatic in early childhood, whereas LHON typically presents between the ages of 15 and 35 years old with rapidly progressive visual loss. The visual prognosis is poor with the majority of individuals eventually fulfilling the legal requirement for blind registration [2]. There are currently no approved disease-modifying treatments available to halt or reverse the loss of retinal ganglion cells. Treatment of IONs is focused on symptom management, genetic counselling, and supporting affected individuals to adapt to low vision through the provision of vision aids and occupational therapy.

In the last 10 years, there has been significant interest in the treatment of inherited ocular disorders by gene therapy, including IONs. Several Phase II and III clinical trials evaluating the effectiveness and safety of gene therapy in individuals who harbour the m.11778G > A mutation responsible for the majority of cases of LHON have recently reached completion. Early results are promising, with a favourable safety profile and a meaningful improvement in visual acuity for study participants. If successful, gene therapy could be a reality for other IONs. Clinical trials for LHON, like other ophthalmic studies, utilise traditional metrics of visual function as study outcome measures, including visual acuity, visual fields, contrast sensitivity, and colour vision [3, 4]. Specific study endpoints vary by study design, but generally involve demonstrating a statistically significant between-group difference for the measure of interest. Assessment of best-corrected visual acuity using the Early Treatment Diabetic Retinopathy Study (ETDRS) chart remains the perceived gold standard for assessing visual function. A three-line (15 letter) change on the ETDRS chart is considered to be clinically significant and an acceptable primary endpoint for clinical trials per the US Food and Drug Administration (FDA).

Although visual acuity remains the most established parameter used by regulatory agencies such as the FDA when considering therapeutic efficacy, it is now recognised that this metric has its limitations as it does not capture the full extent of an individual’s lived experience of their condition. There is increasing awareness of the importance of the patient voice in ophthalmology, with a move away from the sole use of traditional metrics of visual function in clinical trials toward inclusion of metrics that matter more to patients [5]. The patient’s perspective of their health status and their experience of illness, disability, and treatment can be assessed using questionnaires known as patient-reported outcomes measures (PROMs). There has been a proliferation of PROMs developed to assess the experience of ophthalmic disorders and their treatments, particularly for use in therapeutic clinical trials, where impact on quality of life (QoL) is a frequently desired outcome measure [5–7]. In order for PROMs to be effective in clinical practice and research, they have to capture the disease characteristics that matter to the patient [5]. Generic PROMs, including generic QoL PROMs may be useful when comparing the health status or views of individuals across different disease groups. However, they may exclude important aspects that are relevant to specific groups of patients, which are better assessed using a condition-specific PROM.

It is unclear if any currently available PROMs are suitable for use in assessing the experience of individuals affected by an ION. The purpose of this systematic review was to identify and comprehensively evaluate studies capturing the experience of adults and children affected by an ION, focusing on PROMs and qualitative studies where the health status and QoL of these individuals have been explored. Specific aims of the review were to: (1) identify all studies quantitatively assessing QoL in individuals with ION, using a PROM instrument; (2) assess the quality of the identified PROM instruments by determining robustness of content development, psychometric properties including validity and reliability, and responsiveness; (3) identify and appraise qualitative studies of individuals with ION; and (4) identify key themes and messages of the experience of individuals with ION in qualitative studies.

## Method

A systematic electronic database search was undertaken using MEDLINE, EMBASE, PsycINFO, CINAHL Plus, and Scopus. The search strategy utilised index (MeSH) terms and free text words and included four concepts: (1) inherited optic neuropathies; (2) quality of life and health status; (3) patient-reported outcome measures; and (4) qualitative research. The search strategy was adapted for use depending on the database [Appendix 1]. A manual search of the reference lists and citations of included papers for articles that may have been missed by the initial electronic database search was also performed. The search strategy did not incorporate any date or language limits. Inclusion and exclusion criteria are summarised in Table 1.

**Table 1:** Study inclusion and exclusion criteria

Inclusion Criteria: • studies that quantitatively assessed the impact of IONs on any domain of QoL • studies that reported on the development, psychometric assessment, or validation of PROMs to assess the impact of IONs on any domain of QoL • studies that qualitatively explored the impact of IONs on any domain of QoL, through the use of focus groups, structured/semi-structured interviews or questionnaires, and/or literature reviews	Exclusion Criteria: • animal studies and experimental science studies • single case reports, editorials, and conference abstracts

*IONs* inherited optic neuropathies; *PROM* patient-reported outcome measure; *QoL* quality of life

Search results were uploaded to Endnote X8 (Thomson Reuters). After removal of duplicates, titles and abstracts of studies retrieved using the search strategy and those from additional sources were screened by one reviewer (BC) to remove irrelevant articles. A second reviewer (SA) cross-checked 300 (22%) of the studies, and Cohen’s Kappa was calculated to determine inter-rater reliability of the two reviewers. Full text articles were obtained for studies that met the inclusion criteria. Abstracts that did not provide sufficient information to make a decision were taken forward for full-text screening, to minimise the risk of missing a potentially relevant article. Two reviewers (BC and TG) then independently performed a full-text screen. Reasons for exclusion at the full-text stage of screening were recorded. A third reviewer (KP) was consulted if there was disagreement between the two reviewers. The two reviewers independently extracted data from studies meeting the inclusion criteria, using a standardised form.

For each included study, study characteristics (publication year, citation, country/region, sample size, PROM instrument(s)) and characteristics of patients for whom the instrument was developed/assessed/validated, were extracted. This included the type(s) of IONs. For qualitative studies, any identified themes and direct patient quotes/extracts were extracted. The psychometric quality of each identified PROM instrument was assessed using previously described quality criteria [8], based on the FDA guidance document and the ‘COnsensus-based Standards for the selection of health Measurement INstruments’ (COSMIN) risk of bias assessment tool [Appendix 2]. The quality of qualitative studies was assessed using the ‘Critical Appraisal Skills Programme’ (CASP) checklist for qualitative studies [9].

Extracted data from both quantitative and qualitative studies was compiled in a database and then presented separately in tabular form. The characteristics of the patient groups studied (sample size, age, sex, ethnicity, type of ION, co-morbidities, treatments) in both types of studies were summarised. For the data extracted from quantitative studies, the PROM instrument names, QoL domains assessed, and the number of questions included for each domain within the instrument were summarised. For each PROM instrument, the quality assessment for each criterion was summarised and a narrative summary of the findings was provided. For qualitative studies, the methodological approach, participant information and inclusion criteria, study location, and summary of the main findings were reported in tabular format. Subgroup analysis based on individual QoL domains was also conducted, by presenting PROMs by QoL domain or construct, and by content coverage, in tabular format.

The protocol was registered with PROSPERO (CRD42020221807).

## Results

**Fig. 1:**
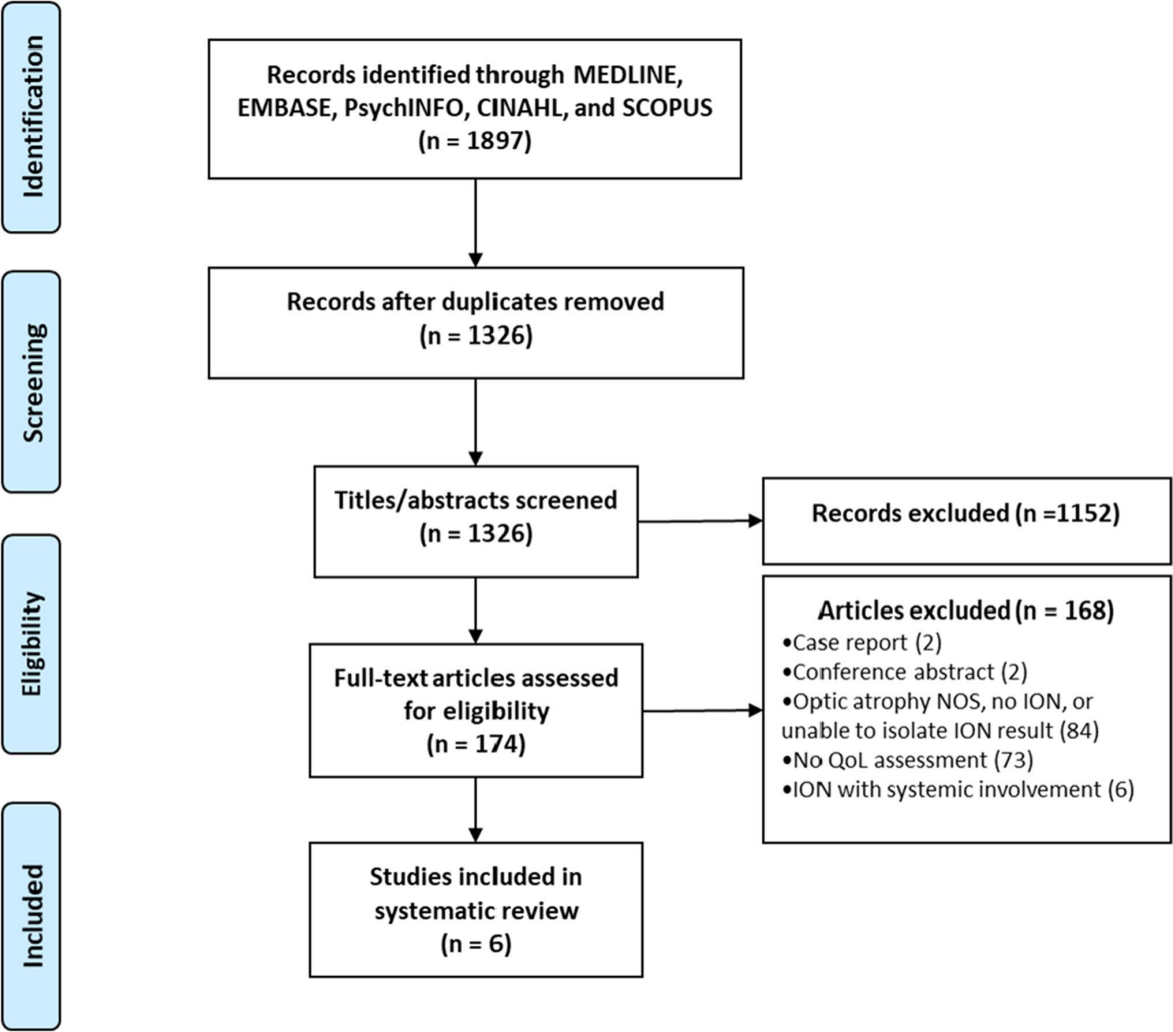
Systematic review flow diagram. ION: inherited optic neuropathy; NOS: not otherwise specified; QoL: quality of life

Figure 1 depicts the flow of information through the different phases of the systematic review. The search conducted on December 1, 2020 identified a total of 1897 studies from MEDLINE (589), EMBASE (1085), PsycINFO (76), CINAHL Plus (16), and Scopus (131). After removal of 571 duplicates, the titles and abstracts of 1,326 studies were screened for eligibility. A sample of 300 studies was cross-checked by the second reviewer, with excellent agreement (99.33% agreement; Cohen’s κ: 0.83) between the two reviewers. One-hundred and seventy-four studies underwent full-text screen. After excluding 168 studies, a total of six studies were included in the review. A manual search of the reference lists of included studies did not yield any additional studies that met eligibility criteria.

### Characteristics of identified studies and study participants

The characteristics of the six included studies are summarised in Table 2 [10–15]. Five studies reported the experiences of individuals with LHON [11–15], and one study focused on the experiences of participants with DOA and ‘DOA + ’ (a clinical syndrome comprising of DOA with extra-ocular manifestations) [10]. One study from a group in the USA involved the development of an online assessment tools for individuals with LHON to retrospectively report and track the emotional impact of their condition using a mixed methods approach [13]. The same group also determined the prevalence of depressive symptoms in individuals with LHON and the impact of their condition on interpersonal relationships and careers, using two self-reported measures in a second study [14]. Two studies reported the vision-related QoL of individuals with LHON and unaffected carriers [11, 15]. One study utilised qualitative methods to describe the lived experiences of seven men affected by LHON living in the UK [12]. The single study involving individuals with DOA and DOA + described vision-related QoL and the prevalence of mood symptoms [10]. The findings of the six included studies are summarised in Appendix 3.

**Table 2:** Characteristics of the six studies included in the systematic revie﻿w

Author (Year)	Country	Sample Size	Type of ION	Age (years)	Disease Duration (years)	Sex	Ethnicity	Co-morbidities	Treatments	Language
Bailie et al. (2013)[10]	UK	30	DOA (confirmed *OPA1* mutation)	Not stated	Not stated	Not stated	Not stated	Not stated	Not stated	English
		8	DOA + (confirmed *OPA1* mutation)							
Cui et al. (2019)[11]	China	55	LHON m.11778G > A affected	Median (IQR): 16.3 (13.9–18.3)	3 months to 3 years from involvement of second eye	49 M: 6F	Chinese	"No concomitant disease that could lead to disability"	Not stated	Chinese
Ferguson & de Abreu (2016)[12]	England, UK	7	LHON (mutation not specified) affected	Range: 21–62	1–43	7 M: 0F	Not stated	Not stated	Not stated	English
Gale et al. (2017)[13]	International: Individual countries not reported	116*	LHON (mutation not specified) affected	Median (range):	Median (range):	88 M: 28F	n(%) White:	n (%) pre-existing mental health problems:	Not stated	English
		28	Emotional 'recovery' group	34.5 (19–70)	9 (0–50)	23 M: 5F	25 (89%)	4 (14.3%)		
		18	Emotional 'unrecovered' group	38 (13–60)	7 (0–38)	11 M: 7F	16 (89%)	2 (11.1%)		
		4	Emotional 'small effect' group	50.5 (17–67)	21 (12–32)	3 M: 1F	3 (75%)	0 (0%)		
Garcia et al. (2017)[14]	Southern California, USA, UMDF mailing list, and social network for LHON individuals	103	LHON (mutation not specified) affected	Mean ± SD: 29.5 ± 13.2	Mean ± SD: 5.0 ± 1.3	80 M: 23F	White: 86 (83.5%) Hispanic: 7 (6.8%) Asian: 6 (5.8%) Pacific Islander: 2 (1.9%) Black/African American: 2 (1.9%)	n (%) pre-existing mental health problems: 8 (7.8%)	Only visual aids stated	English
Kirkman et al. (2009)[15]	UK, The Netherlands, and Germany	132	LHON m.11778G > A: affected	Mean ± SD (range): 43.3 ± 16.9 (13–82)	Mean ± SD: 15.5 ± 15.4	146 M: 50F	All white - except one Asian	Not stated	Not stated	English, Dutch, German
		35	LHON m.3460G > A: affected							
		29	LHON m.14484 T > C: affected							
		138	LHON m.11778G > A: unaffected	Mean ± SD (range): 47.8 ± 14.9 (14–83)	Not applicable	60 M: 146F				
		36	LHON m.3460G > A: unaffected							
		32	LHON m.14484 T > C: unaffected							

*DOA* dominant optic atrophy; *DOA* + ‘dominant optic atrophy plus’ syndrome; *F* female; *LHON* Leber hereditary optic neuropathy; *ION* inherited optic neuropathy; *IQR* interquartile range; *LHON* Leber hereditary optic neuropathy; *M* male; *n (%)* number (percentage); *SD* standard deviation; *UMDF* United Mitochondrial Disease Foundation

^*^116 participants recruited and 81 participants attempted the tool. Only 50 included in analysis after excluding participants who provided fewer than two data points and those whose data points remained the same

Five hundred and two participants, across four of the identified studies, were recruited from the UK, the Netherlands, Germany, and China [10–12, 15]. The two US studies recruited 219 participants internationally through social media and the mailing lists of LHON patient advocacy groups [13, 14]. A breakdown of the location of participants was not provided in these two studies. Consistent with the male predominance of affected individuals, most participants (*n* = 430) in the five studies of individuals with LHON were male. Except for one study of 55 Chinese individuals affected by LHON [11], the majority of participants (*n* = 534) were White. The average age of participants was between 30 and 50 years, although a range of ages was represented. The duration of disease was not uniformly reported in all studies, but included recently diagnosed individuals and those with chronic disease. Three studies reported the genetic mutation of participants [10, 11, 15].

### Identified PROMS and quality assessment

A total of five PROMs used with individuals with ION were identified (Table 3). The Visual Function Index (VF-14), an instrument that measures vision-related activity limitation, was used by three studies to report vision-related QoL [10, 11, 15]. Three instruments were used as measures of emotional wellbeing: the Hospital Anxiety and Depression Scale (HADS); a ‘novel graphical online assessment tool’ (NGOAT); and a measure informed by the Diagnostic and Statistical Manual of Mental Disorders V (DSM-V) criteria for Major Depressive Disorder (MDD). The HADS, a 14-item scale comprised of a 7-item anxiety domain and a 7-item depression domain, was used in the single study of individuals with DOA “to quantify the functional impact of DOA on patients’ QoL” [10]. The NGOAT is a single item instrument developed to allow participants with LHON to retrospectively report their emotional response to vision loss on a 10-point rating scale [13]. The DSM-V criteria for MDD [16], was used to guide the domains of a 9-item measure for assessing the psychological well-being of individuals with LHON [14]. The impact of disease on social wellbeing and work/productive activity was assessed in the same study of individuals with LHON using a 1-item interpersonal interactions and 1-item career goals ‘impact rating’ on a 21-point scale [14].

**Table 3:** Quality assessment of patient-reported outcome measures (PROMs) utilised in studies of individuals with inherited optic neuropathies

Instrument	Domains Concepts Assessed (No. of Items)	Response System	Author (Year)	Instrument Development						Instrument Property				
				Pre-study hypothesis & intended population	Face validity	Item identification	Item selection	Unidimensionality	Response scale	Convergent validity	Discriminant validity	Predictive validity	Test–retest validity	Responsiveness
VF-14	Activity Limitation: Vision-dependent function (14)	Polytomous: 5 options	Bailie et al. (2013)[10]	NA	NA	NA	NA	NA	NA	√√	X	X	X	X
			Cui et al. (2019)[11]	NA	NA	NA	NA	NA	NA	X	X	X	X	√
			Kirkman et al. (2009)[15]	NA	NA	NA	NA	NA	NA	X	X	X	X	X
HADS	Emotional Well-being Anxiety (7) Depression (7)	Polytomous: 4 options	Bailie et al. (2013)[10]	NA	NA	NA	NA	NA	NA	√	X	X	X	X
NGOAT	Emotional Well-being: "Extent of sadness" (1)	Polytomous: 10 point-scale	Gale et al. (2017)[13]	√√	X	X	X	X	√	X	X	X	X	√
DSM-V Criteria for MDD	Emotional Well-being: Symptoms of depression and anhedonia (9)	Dichotomous	Garcia et al. (2017)[14]	√√	√√	X	X	X	X	X	X	X	X	X
Impact Rating	Social Well-being: Interpersonal impact rating (1)	Polytomous: 21-point scale	Garcia et al. (2017)[14]	√√	√√	X	X	X	√√	X	X	X	X	X
	Work/Productive Activity: Career impact rating (1)													

*DSM-V* Diagnostic and Statistical Manual of Mental Disorders; *HADS* Hospital Anxiety and Depression Scale; *MDD* major depressive disorder; *NA* not applicable; *NGOAT* novel graphical online assessment tool; *VF-14* Visual Function Index

None of the included studies provided sufficient information to enable comprehensive assessment of the psychometric quality of the five identified PROMs (Table 3). The three studies utilising pre-existing PROMs (VF-14 and HADS) did not justify the selection of the instruments used [10, 11, 15]. The performance of the VF-14 was compared to the visual acuity of participants in two studies (convergent validity) [10, 11]. One study found a moderate correlation between logMAR visual acuity and VF-14 scores, that is, that as visual acuity worsened in individuals with DOA or DOA + , the lower their VF-14 scores (indicating worsening vision-related activity limitation) [10]. The same study also found a moderate correlation between HADS and VF-14 scores. The study involving Chinese individuals with LHON found an inconsistent correlation between VF-14 scores and visual acuity at different time points, but did not report a correlation co-efficient [11]. The same study detected clinically important changes in VF-14 scores over their study period (responsiveness). Other instrument properties such as discriminant validity, predictive validity, and test–retest reliability could not be determined.

The two studies that described the development of the NGOAT [13], and utilised the DSM-V criteria for MDD [14] and ‘impact rating’ PROMs [14], provided a clear statement of the aims of the outcome measures. The items appeared relevant to the intended population (face validity). However, individuals with ION were not involved in identifying or selecting the items. The authors of the NGOAT study also acknowledged that the ambiguous wording of the single item in their PROM, “to what extent have you felt sad?”, invalidated any ‘advanced’ quantitative analysis of the data they had planned. Instead, the pattern of change in NGOAT scores before, during, and after vision loss was qualitatively analysed with the comments that participants provided. The instrument appeared to have some responsiveness to change, but the data did not allow measurement of a minimally important difference. There was no justification for the response categories used, including the 10-point scale for the NGOAT or 21-point scale for the ‘impact rating’ PROMs. The ‘impact rating’ PROMs also showed significant skew and floor effects. The psychometric performance of the NGOAT, the PROM based on the DSM-V criteria for MDD, and the ‘impact rating’ PROMs, were not reported and could not be determined from the information provided.

### Qualitative studies and quality assessment

The development of the NGOAT to retrospectively assess emotional response to vision loss in individuals with LHON used a mixed methods approach [13]. Participants were asked to justify the rating scores they had indicated on the scale. These responses were analysed qualitatively, focusing on factors that affected mood and adjustment, and recovery for participants. These were further analysed for themes and patterns. However, the qualitative methodology was not discussed and there was no acknowledgement of the reflexivity of the researchers when analysing the collected data. The study found that the extent and duration of sadness was considerably worse when vision loss resulted in loss of relationships, independence, and work or college (university) training. Recovery of mood was also related to these factors; with retraining, new employment, career development, financial security, development of a relationship and marriage, having children, visual aid technology, and sports and travels, all cited as factors associated with recovery of mood. Vulnerability factors in the form of severe hardships such as bereavement, drug or alcohol misuse, academic underachievement, and unplanned parenthood, were observed in participants with long periods of sadness or no recovery of mood after vision loss.

The single qualitative study identified in the review used semi-structured interviews informed by interpretative phenomenological analysis principles to examine the lived experiences of seven men affected by LHON in the UK [12]. One of the study authors had LHON himself and recognised the effect this could have in collecting and analysing the data. This was acknowledged in the study methodology. The study identified six key themes: (1) loss of vision resulted in psychosocial losses including loss of social/communication skills, loss of independence and freedom; (2) participants demonstrated a determination and resolve that enabled them to continue with their lives and overcome challenges; (3) practical methods to adjust to life without vision required developing new skills and using other sensory tools to obtain information; (4) the level of residual vision that remained post LHON-onset appeared to influence participants’ decision to display their blind identity; (5) clarity of a participant’s visual field influenced how they viewed regaining their independence; and (6) all participants acknowledged they experienced moments that evoked a feeling of loss of their sight. Using the CASP checklist, the overall quality of the identified study was excellent (Appendix 4). The only potential source of bias was the study recruitment strategy, which involved advertising the study through two social networking websites and by directly emailing individuals registered with an LHON website. The study authors recognised that it would be erroneous to generalise the results of the study across the LHON population because of the participants that were recruited [12].

## Discussion

There is a paucity of studies reporting the experiences of individuals with ION. This systematic review identified six studies capturing the experiences of individuals with ION, using five PROMs: two pre-existing PROMs not developed for use with individuals with ION per se (VF-14 and HADS) and three new measures for use by individuals affected by LHON (NGOAT for assessing emotional response to vision loss, a measure based on the DSM-V criteria for MDD, and an interpersonal interactions and career goals ‘impact rating’). The studies found that individuals with ION experienced limitations in their activity as a consequence of their vision loss. Many participants also reported symptoms of anxiety and depression, with some individuals affected by LHON satisfying DSM-V criteria for major depression. There was also considerable impact on interpersonal relationships and career.

In research involving individuals with ophthalmic conditions, PROMs should be comprehensive to assess a holistic impact in QoL and include content that is important to the patient group utilising the PROM. Quality of life is a broad, multi-dimensional concept defined by the World Health Organisation as “an individual’s perception of their position in life in the context of the culture and value systems in which they live and in relation to their goals, expectations, standards and concerns” [17]. In ophthalmology, a set of ten QoL domains have been identified previously as being important to patients with ophthalmic conditions including: activity limitation, mobility, visual symptoms, ocular surface symptoms, general symptoms, emotional well-being, social participation, economic, health concerns, and convenience [18, 19]. It is unclear if these domains are relevant or important to individuals with ION. Ocular surface symptoms, for example, are not a feature of IONs, but much more common in diseases such as dry eye syndrome or blepharitis. Despite QoL being a key concept of interest in three of the studies identified in the systematic review, the PROMs that were selected or developed by the researchers primarily focused on activity limitation and emotional well-being. Individuals with ION were not involved in the selection or design of the PROMs identified.

In the literature, constructs like vision-related activity limitation and vision-related QoL are frequently merged with visual function when discussing the patient experience of having an ophthalmic disorder [7, 17], resulting in the selection of PROMs that may not be appropriate for the concept of interest. The VF-14, which was used as a measure of vision-related QoL in three studies [10, 11, 15], was originally designed for patients undergoing cataract surgery and is a widely-used and validated tool that quantifies vision-related activity limitation [20]. Despite this, the VF-14 has been used in clinical trials as a measure for vision-related QoL, including a therapeutic clinical trial for idebenone in individuals with LHON, which did not find a statistically significant difference between the active treatment and placebo groups using the VF-14 [21]. Similar issues have been identified in ophthalmic disease-specific PROMs. A systematic review in 2017 of retina-specific PROMs for retinal diseases such as age-related macular degeneration and diabetic retinopathy, found that existing retina-specific PROMs primarily measured activity limitation or mobility [6].

Factors such as the age and demographic background of the individuals using the PROM also need to be taken into consideration when selecting or developing a PROM. Items in a PROM must be relevant to the group that is utilising the PROM. Although the VF-14 shows high internal consistency and is a reliable, valid instrument for measuring vision-related activity limitation, several items such as difficulty “doing fine handwork like sewing, knitting, crocheting and carpentry” and difficulty “playing games such as bingo, dominos, card games or mahjong” are more relevant to older individuals with cataracts, than young individuals affected by an ION [20]. Similar issues may also occur when using PROMs developed for individuals with low vision. PROMs such as the Veterans Affairs Low-Vision Visual Functioning Questionnaire (VF-VFQ-48) and the Impact of Vision Impairment Profile (IVI), are often developed with the input of individuals affected by glaucoma and age-related macular degeneration, both age-related disorders which are more prevalent in the older population [22, 23]. Individuals affected by an ION are likely to have different needs to those of older patients with low vision.

Impact of LHON on interpersonal relationships and career was measured in one study [14], but only a single item was used to assess the impact of LHON in each of these social participation domains. The majority of participants in this study indicated the lowest possible score (indicating a negative impact). In contrast, the studies that used a qualitative or mixed methods approach, obtained rich detail regarding the impact of LHON on interpersonal relationships and career/education, and the factors that impacted on their emotional response to vision loss. Importantly, both studies identified factors that influenced how individuals with ION responded to these challenges, including disease-related factors, personal factors and attitudes, and external/environmental factors. These factors are consistent with those identified by Albrecht and Devlieger [24], in their ‘balance theory’ explanation for the disability paradox, the observation that many individuals with serious and persistent disabilities actually report that they experience a good or excellent QoL, despite their poor health status or disability. They theorised that the experience of well-being and life satisfaction (good QoL) was contingent on achieving balance between body, mind, and spirit in the context of the larger environment. Factors contributing to good QoL included a ‘can-do’ approach to life and belief that one still had control over their bodies, minds, and lives. For some individuals with disability that they interviewed, disability served to clarify and re-orient their lives. Factors contributing to poor QoL included pain, fatigue, a sense of hopelessness, loss of control of corporal or mental activities, and/or not having a clear purpose or spiritual outlook in life [24].

A key strength of the systematic review was its comprehensive design, which involved multiple databases including those focused on behavioural and social science research and literature, and the inclusion of studies utilising qualitative methodology to gain a more holistic understanding of the experiences of individuals affected by an ION. Despite this, very few studies were identified. Studies, particularly clinical trials, involving individuals with rare disease are often not completed or published [25]. Several reasons for this include lack of funding, difficulty recruiting sufficient patient numbers, and the challenges associated with publishing studies with negative results. Despite the importance of qualitative research addressing concepts that are difficult to quantify numerically such as QoL, feelings, or opinions, only one study utilising qualitative methods was identified in the systematic review. Qualitative research is significantly under-represented in ophthalmology journals. A study in 2017, found that despite 11% of studies identified on the UK Clinical Research Network portfolio utilising qualitative research methods, 0.3% of original research published in three major ophthalmology journals included qualitative methods during the time period screened [26]. Another limitation is the predominance of white men participating in the included studies, which impacts on the generalisability of the studies’ findings. Further qualitative studies are vital to understand the experience of living with an ION, and could inform the content development of an ION-specific PROM. Although individuals with DOA and LHON share similar demographic features such as age of onset, studies identifying similarities and differences in the experiences of individuals with both conditions are required. Given the challenges of developing PROMs for rare diseases, there may be an opportunity to develop a PROM that can also be utilised by individuals with inherited retinal disorders that share similar clinical features, including pattern of visual field impairment.

In conclusion, this systematic review found that the PROMs currently used to report the experiences of individuals affected by an ION are limited in content coverage. This arises from problematic selection of pre-existing PROMs or screening tools that are not intended as measures of QoL for this population, and problems with the content development of new measures. Relying on PROMs such as the VF-14 as a clinical trial endpoint for reporting QoL is highly problematic as the items do not comprehensively cover the QoL domains that are important to individuals with ION. Additionally, the psychometric properties of the identified PROMs were not uniformly reported, calling into question the validity of these instruments for use with the ION population. There is a need to develop an ION-specific PROM for individuals with DOA and LHON to report their experiences of living with their condition, that can be used in future clinical trials and natural history studies. The psychometric properties of the PROM also need to be evaluated in the development of the PROM, to ensure that the findings are robust when used in studies involving individuals with ION.

## Supplementary Information

Below is the link to the electronic supplementary material.

Supplementary file1 (DOCX 26 KB)

## Data Availability

The authors confirm that the data supporting the findings of this study are available within the article and its supplementary materials.
