# Supplementary material for: Capturing the experiences of patients with inherited optic neuropathies: a systematic review of patient-reported outcome measures (PROMs) and qualitative studies
Source: Graefes Arch Clin Exp Ophthalmol. 2022 Jan 13;260(6):2045–55. doi: 10.1007/s00417-021-05534-0 (PMC9061690; doi:10.1007/s00417-021-05534-0)
Supplement: Supplementary file 1 — Supplementary file1 (DOCX 26 KB) [file 417_2021_5534_MOESM1_ESM.docx]

**APPENDICES**

**Appendix 1. Search strategy**

Database: Embase <1974 to 2020 November 30>

1 exp optic atrophy/ (11016)

2 optic atrophy.tw. (5557)

3 ((inherited or hereditary or genetic) adj2 optic).tw. (2351)

4 (leber or lhon or doa or adoa or kjer or opa1).tw. (6183)

5 or/1-4 (16219)

6 exp "Quality of Life"/ (511256)

7 exp "Activities of Daily Living"/ (92537)

8 exp "Attitude to Health"/ (114830)

9 exp health status/ (247622)

10 exp health status indicators/ (31742)

11 exp "Value of Life"/ (393888)

12 exp patient satisfaction/ (142530)

13 or/6-12 (1366885)

14 ("quality of life" or qol or hrqol or hrql or "activities of daily living" or "activity of daily living" or ADL$ or "health status" or "impact of" or "lived experience" or well-being or "well being" or "patient satisfaction" or "visual function" or "functional vision" or symptom* or psychosocial or psycho-social or psychological or identity or coping or adapt* or adjustment or depress* or anxiety or worry or distress or discrimination or stigma or disab*).tw. (5436397)

15 13 or 14 (6084777)

16 exp patient reported outcome measures/ (26674)

17 exp "Surveys and Questionnaires"/ (740016)

18 exp interview, psychological/ (871)

19 exp health surveys/ (228203)

20 exp qualitative research/ (82541)

21 exp "Grounded Theory"/ (7799)

22 interview/ (213427)

23 "Personal Narrative"/ (39373)

24 or/16-23 (1209914)

25 (pros or pro or proms or patient-reported or "patient reported" or patient engage* or measure* or scale or index or instrument or "assessment tool" or interview* or questionnaire$ or survey or "focus group" or framework or theor* or model or dimension or concept$ or "grounded theory" or "narrative analysis" or "thematic analysis" or phenomenologic* or ethnograph* or meta-ethnograph*).mp. (12590015)

26 24 or 25 (12632816)

27 5 and 15 and 26 (1085)

**Appendix 2. Criteria used to assess the quality of patient-reported outcome measures[8]**

| **Property** | **Definition** | **Quality Criteria** |
| --- | --- | --- |
| **Instrument development** | | |
| Pre-study hypothesis and intended population | Specification of the hypothesis pre-study and if the intended population have been studied | ✓✓- Clear statement of aims and target population, as well as intended population being studied in adequate depth ✓- Only one of the above or generic sample studied X- Neither reported |
| Actual content area (face validity) | Extent to which the content meets the pre-study aims and population | ✓✓- Content appears relevant to the intended population ✓- Some relevant content areas missing X- Content area irrelevant to the intended population |
| Item identification | Items selected are relevant to the target population | ✓✓- Evidence of consultation with patients, stakeholders and experts (through focus groups/one-to-one interview) and review of literature ✓- Some evidence of consultation X- Patients not involved in item identiﬁcation |
| Item selection | Determining of ﬁnal items to include in the instrument | ✓✓- Rasch or factor analysis employed, missing items and ﬂoor/ceiling effects taken into consideration. Statistical justiﬁcation for removal of items ✓- Some evidence of above analysis X- Nil reported |
| Unidimensionality | Demonstration that all items ﬁt within an underlying construct | ✓✓- Rasch analysis or factor loading for each construct. Factor loadings >0.4 for all items ✓- Cronbach’s alpha used to determine correlation with other items in instrument. Value >0.7 and <0.9 X- Nil reported |
| Response scale | Scale used to complete the measure | ✓✓- Response scale noted and adequate justiﬁcation given ✓- Response scale with no justiﬁcation for selection X- Nil reported |
|  |  |  |
| **Instrument performance** | | |
| Convergent validity | Assessment of the degree of correlation with a related measure | ✓✓- Tested against appropriate measure, Pearson’s correlation coefﬁcient between 0.3 and 0.9 ✓- Inappropriate measure, but coefﬁcient between 0.3 and 0.9 X- Nil reported or tested and correlates <0.3 or >0.9 |
| Discriminant validity | Degree to which an instrument diverges from another instrument that it should not be similar to | ✓✓- Tested against appropriate measure, Pearson’s correlation coefﬁcient <0.3 ✓- Inappropriate measure, but coefﬁcient <0.3 X- Nil reported or tested and correlates >0.3 |
| Predictive validity | Ability for a measure to predict a future event | ✓✓- Tested against appropriate measure and coefficient >0.3 ✓- Inappropriate measure but coefﬁcient >0.3 X- Nil reported or tested and correlates <0.3 |
| Test-retest reliability | Statistical technique used to estimate components of measurement error by testing comparability between two applications of the same test at different time points | ✓✓- Pearson’s r value or ICC >0.8 ✓- Measured but Pearson’s r value or ICC <0.8 X- Nil reported |
| Responsiveness | Extent to which an instrument can detect clinically important differences over time | ✓✓- Discussion of responsiveness and change over time. Score changes > MID over time ✓- Some discussion but no measure of MID X- Nil reported |

✓✓-positive rating, ✓-acceptable rating, X-negative rating.

ICC, intraclass coefficient; MID, minimally important difference.

**Appendix 3. Summary of findings from included studies**

| **Author (Year)** | **Summary of Study** |
| --- | --- |
| Bailie et al. (2013)[10] | In this study, the functional impact of DOA and DOA+ on patients' quality of life was measured using the VF-14 and HADS. Visual acuity data was obtained from patients' records. Mean VF-14 for entire cohort was 37.3 ± 25.6. DOA+ patients had significantly VF-14 lower scores than DOA patients, and significantly higher depression scores (but not anxiety scores). Borderline or definite symptoms of anxiety and depression were present in 19/38 (50.0%) and 7/38 (18.4%) of the entire cohort, respectively. There was a statistically significant correlation between VF-14 score and logMAR vision, anxiety score, and depression score. |
| Cui et al. (2019)[11] | In this longitudinal study, the quality of life of Chinese LHON patients with the m.11778G>A mutation was assessed prospectively using the VF-14 at six months, one year, and three years after involvement of the second eye. VF-14 scores were 18.0 ± 19.2, 19.9 ± 20.0, and 20.7 ± 20.2, respectively. There was a significant difference in the score at one year compared to six months follow up, and in three years compared to one year follow up. Logistic regression analysis test showed that the VF-14 scores at six months significantly correlated with visual acuity, but not age of onset. Improvement in VF-14 score significantly correlated with age of onset, but not with visual acuity. There was no significant correlation between visual acuity and age of onset. Tasks that showed significant improvement between six months and one year included: 'seeing steps, curbs, or stairs', 'doing handiwork', 'playing sports', 'cooking', and 'watching television'. Tasks that showed significant improvement between one year and three years included: 'cooking' and 'watching television'. |
| Ferguson & de Abreu (2016)[12] | The six key themes illustrating participants lived experience of LHON in this qualitative study were: (1) psychosocial losses; (2) attitudes and coping strategies; (3) development of practical skills; (4) identity; (5) regaining independence; and (6) recurrent loss.  (1) Loss of vision, a sensory tool affecting a person's capacity to live and within the world, resulted in psychosocial losses including loss of social/communication skills, loss of independence and freedom. Challenges of living with LHON were reflected in their emotional experience. Daily hassles and associated losses could always culminate frustration.  (2) Participants demonstrated a determination and resolve that enabled them to continue with their lives and overcome challenges. Humour was used by some to reframe embarrassing or frustrating moments. (3) Practical methods to adjust to live without vision required developing new skills and using other sensory tools to obtain information eg. assistive technologies, touch typing using sound or touch. Meeting other visually impaired people inspired some to overcome barriers around their abilities and try new things (4) The level of residual vision that remained post LHON onset appeared to influence the decision to display their blind identity. Across the discourse on identity was the public's image of a 'blind person' and reactions to symbols of this perception eg. white cane. Overtly displaying a blind identify places the individual into a broad category of blind people that doesn't accurately represent the participant's skills, abilities, and attitudes.  (5) Clarity of a participant's visual field influences how they viewed regaining their independence. For some, practical techniques allowed them to live and work independently. For others, the notion of regaining independence was focused on placing themselves in unfamiliar situations to gain confidence in their abilities as a blind person. For those whose visual field was most affected, regaining independence meant becoming reliant on other tools for information eg. using the white cane. (6) All participants acknowledged they experienced moments that evoked a feeling of loss for their sight. Practical and emotional coping mechanisms permit the individual to overcome barriers and lead a fulfilling life. However, limited sight means that they have fewer options in life and reduced visual experience of a moment, leading to intermittent feelings of loss and frustration reminiscent of their early period of adjustment. |
| Gale et al. (2017)[13] | In this prospective study with international online participation, a new tool was developed to study emotional response to bilateral vision loss graphically over time. Participants were asked to indicate the extent that they felt sad on a 10-point Likert scale over a time period extending from 10 years prior to diagnosis to the current year, and providing a comment explaining their selection. Response rate was 43%. After excluding participants who provided fewer than two data points or whose data points were all the same, three groups emerged: those whose mood had decreased around the time of diagnosis and then recovered; those whose decrease in mood did not recover; and those for whom vision loss did not seem to be a major influence on mood compared with other life events. Factors related to recovery from initial sadness from vision loss included work, education, romantic relationships, and family. Loss of relationships, independence, and work or college training was associated with extent and duration of sadness. After visual loss, 33% of participants in the recovered group, 47% in the unrecovered group, and 50% of the small effect group had a psychologic consultation. |
| Garcia et al. (2017)[14] | In this prospective study, an online questionnaire was completed by individuals with LHON to evaluate the effects of profound vision loss on psychological wellbeing with regard to mood, interpersonal interactions, and career-oriented goals. The online questionnaire was developed from the DSM-V criteria for MDD (yes/no responses). Additionally, participants were asked to appraise the effects of vision loss on the quality of their interpersonal interactions and career goals on a 21-point Likert-type scale. Participants were also asked about psychological supports and use of electronic visual aids. 51/103 (49.5%) participants met criteria for presumed depression after vision loss, without any significant difference between males and female participants. 73/102 (70.9%) participants selected a negative interpersonal IR; 17/103 (16.5%) indicated a positive interpersonal IR; and 13/103 (12.6%) indicated a neutral interpersonal IR. The median interpersonal IR was ─ 5. 76/103 (73.8%) indicated a negative career IR; 23/103 (22.3%) indicated a positive career IR; and 4/103 (3.9%) indicated a neutral impact on career. Both ratings were significantly worse for depressed versus non-depressed participants. Older age at diagnosis corresponded to higher prevalence of depression and increased incidence of negative interpersonal IR and career IR. 68% of participants used electronic visual aids; controlling for age, social well-being index was higher among these individuals than for those who did not use electronic aids. 52.4% asserted that they derived emotional support from their ophthalmologist. |
| Kirkman et al. (2009)[15] | In this study, visual disability in affected and unaffected individuals with molecularly confirmed LHON was assessed using the VF-14. The mean VF-14 score was 25.1 ± 20.8 in affected patients, compared with 97.3 ± 7.1 in unaffected carriers. Within affected patients, patients with the m.14484T>C mutation had significantly higher score compared with those carrying the m.3460G>A mutation and m.11778G>A mutation, but there was no significant correlation between VF-14 score and disease duration. The mean VF-14 score was 23.4 ± 19.1 among affected individuals with disease duration ≤1 year and 25.3 ± 21.0 among affected individuals with disease duration > 1 year. 'Reading small print' and 'reading a newspaper or book' were the two activities of daily living that caused the greatest subjective difficulty. |

**Appendix 4. Quality assessment of Ferguson & de Abreu (2016) [12] article, using Critical Appraisal Skills Programme (CASP) checklist for qualitative studies**

| **Screening Questions** | |  | **Yes** | **Can't Tell** | **No** |  | **Comments** |
| --- | --- | --- | --- | --- | --- | --- | --- |
| 1. | Was there a clear statement of the aims of the research? |  | X |  |  |  | Current LHON research focuses on its biology, but provides no insight into the psychological impact of the disease. Aim was to hear the voices of people living with LHON to examine their lived experience. |
| 2. | Is a qualitative methodology appropriate? |  | X |  |  |  | Yes, a qualitative method was appropriate given the aims of the research to examine the lived experience of people living with LHON. |
|  |  |  |  |  |  |  |  |
| **Detailed Questions** | |  |  |  |  |  |  |
| 3. | Was the research design appropriate to address the aims of the research? |  | X |  |  |  | Narrative semi-structured interviews informed by Interpretative Phenomenological Analysis (IPA) principles was deemed to be the most appropriate style of analysis because the research was aimed at explicating the essential meaning the participants were making of their conscious worlds. Justification for using IPA principles of analysis was also provided. |
| 4. | Was the recruitment strategy appropriate to the aims of the research? |  |  |  | X |  | Participants were recruited through two social networking sites and by directly emailing people registered with an LHON website. This may have biased potential participants to those who are actively involved in the LHON online community. |
| 5. | Was the data collected in a way that addressed the research issue? |  | X |  |  |  | Narrative semi-structured interviews were conducted with an interview schedule that began with an open question allowing participants to describe their experience in their own words, before returning to specific topics to explore their meaning in greater detail. |
| 6. | Has the relationship between researcher and participants been adequately considered? |  | X |  |  |  | The field researcher and one of the study authors was identified as having LHON, which aided their ability to explore participants' narratives and to appreciate the basic assumptions about medical, social, and personal experiences within the participants' discourse. However, this personal experience with LHON was also recognised as a potential pitfall within the analytical process, and the second author moderated the process. |
| 7. | Have the ethical issues been taken into consideration? |  | X |  |  |  | Institutional ethical approval was obtained prior to interview. Informed consent was obtained from all participants. Participants were all given a brochure for emotional support service prior to the interview, in preparation for any distress that may have been caused by the discourse. Interviews were digitally recorded and fully transcribed. To protect anonymity, pseudonyms were used in the published research article. |
| 8. | Was the data analysis sufficiently rigorous? |  | X |  |  |  | Clear description of the IPA approach is provided. Recognition of one of the authors introducing bias (See Question 6.) and approach to counter this. |
| 9. | Is there a clear statement of findings? |  | X |  |  |  | Findings are explicit and discussed in relation to the original research questions. There is also adequate discussion of different viewpoints, including explanations for these viewpoints. Limitations of the study are discussed in a separate paragraph, including the possible influence the author with LHON had on data collection and potentially in the analysis. |
| 10. | How valuable is the research? |  |  |  |  |  | The authors recognised that it would be erroneous to generalise the results of this study across the LHON population because of the participants that were recruited. Recommendations for future research that would overcome this bias are discussed. The implications of the study findings at a broader practice or policy level are not discussed. |
